# Incidence of Symptomatic Gallstone Disease in Bariatric Patients Undergoing Sleeve Gastrectomy, and the Dilemma of Prophylactic Cholecystectomy: A Single-Center Retrospective Study

**DOI:** 10.7759/cureus.87243

**Published:** 2025-07-03

**Authors:** Mazin A Alsheikhly, Ahmed Alsheikhly, Mohammed Humeid, Amna M Albu Mahmud, Maricar A Rulloda-Agoncillo

**Affiliations:** 1 General Surgery, Hamad General Hospital, Doha, QAT; 2 Emergency Medicine, Hamad General Hospital, Doha, QAT; 3 Trauma, Hamad General Hospital, Doha, QAT

**Keywords:** bariatric surgery, biliary colic pain, gallbladder, gallstone disease, laparoscopic sleeve gastrectomy (lsg), laporoscopic cholecystectomy, post-sleeve gastrectomy, weight loss and obesity

## Abstract

Introduction

The global obesity "pandemic" has resulted in an increase in bariatric surgeries, with laparoscopic sleeve gastrectomy (LSG) becoming the most commonly performed procedure. While rapid weight loss following LSG offers health benefits, it also raises the risk of gallstone formation. This retrospective study aimed to identify the prevalence of symptomatic gallstone disease after sleeve gastrectomy, determine the incidence of cholecystectomy, and evaluate the advantages of prophylactic cholecystectomy in a Qatari population.

Methodology

The study reviewed the charts of patients who underwent LSG at Hamad General Hospital, Doha, Qatar, between January and December 2021, with follow-up extending to May 2025. A total of 105 patients underwent LSG during this period. After excluding those with prior cholecystectomy and asymptomatic cases, 98 patients remained in the cohort.

Results

Among the patients in the cohort, 16 patients (16.3%) developed symptomatic gallstone disease. The cohort consisted of 64 female patients (61.0%) and 41 male patients (39.1%), with a mean age of 29.1±12.7 years. Female patients accounted for 81.2% of gallstone cases, while males accounted for 18.8%. Patients who developed gallstones were younger (median 21.5 years) than those who did not (median 26.0 years), though this difference was not statistically significant (p=0.237). The most compelling finding was the relationship between weight loss rate and the timing of gallstone diagnosis. A significant negative correlation was observed (r=-0.553, p=0.0262), indicating that faster weight loss was associated with earlier gallstone development. The Kruskal-Wallis H-test showed that patients with early diagnosis (≤6 months) had significantly higher weight loss rates (median 12.68 kg/month) compared to the intermediate (3.72 kg/month) and late diagnosis groups (2.21 kg/month) (p=0.0045). A Post-Hoc test was conducted among these groups, confirming that the early diagnosis (≤6 months) group had a significantly faster rate of weight loss compared to both the intermediate and late diagnosis groups. Linear regression analysis revealed that the weight loss rate explained approximately 30% of the variance in diagnosis timing.

Conclusion

One major postoperative complication after LSG is symptomatic gallstone disease. Its clinical relevance is highlighted by the study cohort's observed incidence rate of 16.3%. Our data suggest that a selective approach, based on individual risk factors for each patient, is warranted, with close follow-up of such patients. The most compelling finding is that the weight loss rate, rather than total weight, is a critical and statistically significant predictor of gallstone formation, especially for early onset, even though female sex remains a strong predisposing factor. This can serve as an important clinical marker for risk stratification and potential prophylactic intervention. A critical window for possible intervention is highlighted by the median time to gallstone diagnosis, which is approximately a year after surgery.

## Introduction

It has been reported that the incidence of obesity is considered to have reached pandemic levels across the globe. As the number of affected individuals continues to rise, it poses a significant public health concern [1]. For those struggling with severe obesity, bariatric surgery has proven to be the most successful treatment option, consistently leading to greater and more sustained weight reduction compared to the most effective non-surgical approaches [2]. Among the various types of bariatric surgery, laparoscopic sleeve gastrectomy (LSG) has become the most widely accepted and frequently performed primary procedure worldwide [3]. Its popularity stems from its comparative ease of execution, effectiveness in achieving substantial weight loss, and ability to improve health issues related to obesity [3].

With the increase in obesity rates globally, the number of bariatric surgeries has also risen, making the study of postoperative complications, such as gallstone disease, increasingly important. Gallstone disease, which includes the formation of gallstones and any related complications, is a well-known and common consequence of bariatric surgery, including LSG [2]. A significant benefit of LSG is rapid weight loss; however, this also increases the risk of developing gallstones in these patients. While many individuals may develop gallstones after LSG, only a few will experience symptoms that necessitate medical or surgical treatment [2].

Risk factors for developing symptomatic gallstones post LSG

Several factors identified in the medical literature may increase the chance of developing symptomatic gallstones after LSG. Some studies suggest that having a higher BMI before surgery might be linked to an increased risk of symptomatic gallstones following bariatric surgery [4]. Additionally, a higher BMI reduction after the operation has been statistically associated with an increased risk of symptomatic gallstones after surgery [5]. Rapid weight loss, in general, is a well-recognized and significant risk factor for developing gallstones after LSG [6]. Being female has also been reported as a risk factor, with female patients showing a higher incidence of gallstones and biliary complications after bariatric surgery [7]. Furthermore, patients who have asymptomatic gallstones before undergoing bariatric surgery are at a greater risk of developing symptomatic disease after the operation [4].

The role of age as a risk factor is less clear. Some studies suggest that older age may protect against symptomatic gallstone disease [8], while others indicate it as a risk factor [4]. The connection between other health conditions and gallstone formation after LSG is not entirely consistent across studies. While some studies have found no significant link between conditions like diabetes, high blood pressure, or high cholesterol and gallstone formation [9], others have reported a higher incidence of gallbladder disease in patients with type 2 diabetes [10]. Finally, having a family history of gallstones has also been identified as a potential risk factor that increases the likelihood of individuals developing gallstones after LSG [11].

Mechanisms underlying gallstone formation after LSG

Rapid and significant weight loss, a primary goal and outcome of LSG, is a well-established major risk factor for developing gallstones [6]. Studies have indicated that losing more than 1.5 kg per week or achieving a total weight loss of 25-30% is linked to a significantly higher risk of gallstone formation [7]. Notably, patients who develop gallstones after LSG tend to lose more weight in the initial months following the surgery compared to those who do not experience this complication [2]. The amount and speed of weight loss appear to be directly correlated with the likelihood of gallstone formation in this patient group.

The rapid weight loss associated with LSG leads to significant changes in bile composition, increasing the likelihood of stone formation. Specifically, there is an increased release of cholesterol into the bile, resulting in a state where the bile contains more cholesterol than it can effectively dissolve. Furthermore, the reduced food intake that is typical after LSG leads to a decreased need for bile in the digestive process. However, the liver continues to produce bile at a relatively constant rate, leading to a higher concentration of bile within the gallbladder. This concentrated bile, now overloaded with cholesterol, is more likely to form gallstones. Rapid weight loss has also been shown to increase the concentration of mucin in the bile, which can further accelerate gallstone formation by creating a thick, gel-like substance that facilitates the formation and growth of cholesterol crystals [7].

Another important factor contributing to gallstone formation after LSG is reduced movement of the gallbladder. The less frequent and smaller amounts of food intake following LSG can weaken the standard nerve signals that generate the gallbladder to contract and empty its contents. This impaired emptying of the gallbladder results in a larger amount of bile remaining in the gallbladder, allowing bile to sit stagnant, both of which create conditions favorable for the formation of gallstones. The infrequent contraction of the gallbladder allows bile to remain in the gallbladder for more extended periods, increasing the likelihood of cholesterol crystallization and stone development [7].

While it is less of a concern in LSG compared to other bariatric procedures, such as Roux-en-Y gastric bypass, the possibility of injury to the hepatic branch of the vagus nerve during surgery remains a consideration [2]. This nerve plays a crucial role in controlling the contraction of the gallbladder. Damage to this nerve impairs the gallbladder's ability to contract effectively, potentially contributing to bile sitting stagnant and an increased risk of gallstone formation [2]. However, the gastrohepatic ligament, through which the hepatic branch of the vagus nerve passes, is usually preserved during LSG. This preservation generally results in a lower risk of vagus nerve injury compared to procedures like Roux-en-Y gastric bypass (RYGB), where the ligament is often divided. Nevertheless, the possibility of vagal nerve injury, although less likely in LSG, cannot be completely ruled out as a contributing factor in some cases of gallstone formation after surgery [7].

Emerging research also suggests a probable role for changes in gut bacteria in the development of gallstones following bariatric surgery. Bariatric surgery leads to alterations in the various bacteria naturally present in the gut, which can, in turn, affect the processing of bile acids. Studies have suggested that certain bacteria, such as *Ruminococcus*, may be linked to a higher risk of gallstone formation following bariatric surgery. Additionally, a higher number of other bacterial groups, such as *Lactobacillaceae* and *Enterobacteriaceae*, may offer some protection against gallstone development. The complex relationship between bile acid processing and natural gut bacteria suggests that the types of bacteria in the gut could be a future target for preventive strategies aimed at reducing the risk of gallstone formation after LSG [7].

There is currently no global consensus on concurrent laparoscopic cholecystectomy (LC) during LSG. However, a significant debate has arisen concerning the role of prophylactic cholecystectomy. This involves removing the gallbladder during LSG in patients who do not already have symptomatic gallstones [3]. Having accurate information on the frequency of symptomatic gallstone disease is essential for making informed medical decisions in the care of bariatric patients [2]. Identifying the risk factors that raise the likelihood of developing symptomatic gallstones after LSG is important, as it can help categorize and identify those who may benefit most from preventive measures or require closer monitoring after surgery [5]. This issue requires careful consideration of the potential advantages of preventing future gallstone-related problems against the risks and costs associated with an additional surgical procedure. The decision to perform prophylactic cholecystectomy is complex because the occurrence of symptomatic disease varies, and the procedure itself has potential benefits as well as drawbacks. High-quality research in this area can lead to a more effective selection of patients for prophylactic cholecystectomy and the development of more personalized care plans following surgery.

No previous study has been conducted in Qatar to explore the incidence of gallstone disease following any bariatric surgery. This study aims to identify the prevalence of symptomatic gallstone disease in patients after sleeve gastrectomy and the incidence of those who underwent cholecystectomy post-sleeve gastrectomy, as well as to evaluate the potential benefits of prophylactic cholecystectomy in this population.

## Materials and methods

This study was conducted in accordance with the guidelines stated in the Declaration of Helsinki. A retrospective cohort chart review was performed on patients who underwent LSG at Hamad General Hospital, Doha, Qatar, between January 2021 and December 2021. A comprehensive review of each patient's electronic medical record (EMR) was carried out. Demographics, surgical details, pre-existing gallstone status, and pre-surgical measurements were collected for every patient. The incidence of patients who underwent post-LSG LC due to gallstone disease was evaluated.

Eligibility criteria and data collection

A list of all bariatric surgeries during that period was extracted on an Excel sheet (Microsoft Coporation, Redmond, Washington, United States), and from this, details of all patients who underwent LSG were gathered. A manual analysis of each case was conducted. All patients documented to have gallstones prior to LSG, those who had LC before LSG, and patients who developed gallstones but remained asymptomatic were excluded. Inclusion criteria were limited to patients with no previous history of gallstones who underwent LSG and developed symptomatic gallstone disease upon follow-up.

The data collected included each patient's age, weight, BMI, admission date, and discharge date. Afterwards, the patient files were reviewed for any presentations related to gallstones, identifying clinical findings, blood work, and ultrasound results that confirmed symptomatic gallstone disease. For the patients who developed symptomatic gallstone disease, the time to gallstone development was noted, along with their weight and BMI, and whether they had undergone LC.

Statistical analysis

The statistical analysis was performed using IBM SPSS Statistics for Windows, version 27 (Released 2019; IBM Corp., Armonk, New York, United States), and the following statistical tests were conducted: Descriptive statistics were used for demographic variables, including mean, median, standard deviation (SD), and interquartile range (IQR). Statistical significance was defined as a p-value of less than 0.05. The Mann-Whitney U test compared non-normally distributed continuous variables between the gallstone and non-gallstone groups. The chi-square test was used to compare categorical variables (gender distribution) between groups. The Pearson correlation coefficient (r) assessed the relationship between weight loss rate and time to gallstone diagnosis. The Kruskal-Wallis H-test compared weight loss rates among the three temporal groups (early, intermediate, and late diagnosis). Post-hoc analysis was conducted using the Pairwise Mann-Whitney U-test with Bonferroni correction to identify which specific groups had statistically significant differences in weight loss rates. Linear regression analysis determined how much of the variance in time to gallstone diagnosis could be attributed to the rate of weight loss. As specified, cases of asymptomatic gallstones were excluded from the statistical analysis to focus on clinically significant disease.

## Results

A total of 105 patients underwent LSG in the study period, with a gender distribution of 64 female (61.0%) and 41 male (39.1%) patients, reflecting the higher prevalence of female patients seeking bariatric surgery. This gender distribution aligns with broader trends in bariatric surgery populations worldwide, where women typically make up approximately 60-80% of patients [9]. The mean age of the cohort was 29.1 ± 12.7 years, with a median of 26 years and a range of 14-61 years (Table 1). The IQR was 19-37 years, indicating that half of the patients fell within this age range. This relatively young average age highlights that severe obesity is impacting individuals early in life, with potential long-term health implications if left untreated.

**Table 1:** Demographics of all patients who underwent LSG in the study period (N=105) LSG: laparoscopic sleeve gastrectomy

Variable	Mean ± SD	Range	Median (IQR)
Age (years)	29.1 ± 12.7	14 - 61	26 (19, 37)
Preoperative Weight (kg)	124.9 ± 23.8	44 - 207	122 (110, 138)
Preoperative BMI (kg/m^2^)	46.2 ± 9.3	35 - 116	45 (42, 48)

An independent t-test was conducted to analyze the preoperative weight by gender. The results, as shown in Table 2, yielded (t = 6.058, p < 0.001) a statistically significant difference in preoperative weight between genders. It was noted that male patients have significantly higher preoperative weights than female patients, with a mean difference of approximately 25 kg.

**Table 2:** Gender-based comparison of preoperative weight in all patients who underwent LSG in the study period (N=105) *24.93 kg higher in male patients; ** Independent t-test LSG: laparoscopic sleeve gastrectomy

Gender	Count	Mean ± SD*	Median	Min-Max	Statistical Test**
Female	64	115.14 kg ± 19.55 kg	113.00 kg	44-195 kg	t = 6.058 p < 0.001
Male	41	140.07 kg ± 22.09 kg	138.00 kg	100-207 kg	

After excluding the patients who had already undergone LC prior to LSG and asymptomatic patients, a total of 98 patients remained in the analysis. Among these, 16 patients developed symptomatic gallstone disease following LSG.

Comparison of continuous variables between patients with and without gallstones

Patients who developed gallstones were younger (median age 21.5 years) compared to those who did not develop gallstones (median age 26 years). Table 3 shows that the p-values for age, preoperative weight, and preoperative BMI are all greater than the standard significance level of 0.05. Thus, these differences did not reach statistical significance.

**Table 3:** Comparison of continuous variables between patients who developed and did not develop gallstones after LSG (N=98) *Mann-Whitney U Test LSG: laparoscopic sleeve gastrectomy

Variable	Patients with gallstones (n=16), median (IQR)	Patients without gallstones (n=82), median (IQR)	U Statistic*	P-value
Age (years)	21.5 (18.75, 27.50)	26.0 (19.00, 38.50)	532.5	0.237
Preoperative Weight (kg)	121.5 (112.75, 127.50)	122.5 (109.25, 138.75)	640	0.882
Preoperative BMI ( kg/m^2^)	45.0 (43.00, 46.25)	45.0 (41.25, 49.00)	646.5	0.931

Gender distribution between patients with and without gallstones

A notable gender disparity was observed, with female patients comprising 81.2% of gallstone cases compared to only 18.8% of males. In contrast, the non-gallstone group had a more balanced gender distribution, with 56.1% female and 43.9% male. Despite this apparent difference, Table 4 shows that the Chi-square Test yielded a p-value of 0.059 (χ² Value: 3.55), which does not meet the conventional threshold for statistical significance. Thus, there is not enough statistical evidence to conclude that there is a significant association between gender and the presence or absence of gallstones.

**Table 4:** Chi-square test for the association of gender and the presence of gallstones P-value: 0.059 (no statistical significance) *Observed (O): The actual counts from the sample (e.g., 13 female patients had gallstones); **Expected (E): The calculated frequencies expected in each category if there were no relationship between gender and gallstones (i.e., if the null hypothesis were true). These values are calculated based on the marginal totals of the rows and columns.

Gender	Group	Observed (O)*	Expected (E)**	(O - E)	(O - E)²	(O - E)² / E
Female	Gallstone	13	9.63	3.37	11.36	1.18
Female	Non-Gallstone	46	49.37	-3.37	11.36	0.23
Male	Gallstone	3	6.37	-3.37	11.36	1.79
Male	Non-Gallstone	36	32.63	3.37	11.36	0.35
Chi-Square Value (χ²)	-	-	-	-	-	χ² = 3.55

Weight reduction at the time of gallstone diagnosis

Weight measurements at the time of gallstone diagnosis revealed significant changes from pre-surgical values, as can be seen in Table 5. Patients with gallstones experienced a mean weight loss of 37.62 ± 12.61 kg, representing a 30.80 ± 9.57% reduction from their initial weight. Similarly, BMI decreased by a mean of 13.75 ± 5.14 kg/m^2^. These substantial changes in weight and BMI highlight the rapid weight loss that typically precedes gallstone formation in this population.

**Table 5:** Weight loss characteristics in patients who developed gallstones after LSG (N=16) lsg: laparoscopic sleeve gastrectomy

Variable	Mean ± SD	Median	Range
Weight at diagnosis (kg)	85.00 ± 16.35	82.00	64.0 - 120.0
Weight loss (kg)	37.62 ± 12.61	35.50	12.0 - 59.0
Weight loss (%)	30.80 ± 9.57	31.51	9.1 - 44.2
BMI reduction (kg/m^2^)	13.75 ± 5.14	13.50	2.0 - 21.0
Time to diagnosis (months)	12.82 ± 9.30	11.30	2.2 - 35.9
Weight loss rate (kg/month)	4.86 ± 5.11	2.78	1.1 - 22.1

Time to development of gallstones

Table 5 shows that the temporal pattern of gallstone development showed considerable variability, with a mean time to diagnosis of 12.82 ± 9.30 months (range: 2.2 - 35.9 months) after LSG. This wide range suggests that while some patients develop gallstones relatively early in their post-surgical course, others may experience delayed onset even years after the procedure. The median time to diagnosis of 11.3 months is particularly noteworthy, as it suggests that the highest risk period may be around the one-year mark post surgery, which coincides with the period when weight loss typically begins to plateau after bariatric procedures. This timing may reflect a critical intersection of factors, including stabilizing weight, changing dietary patterns, and adaptation of gallbladder function.

Weight reduction from LSG to gallstone diagnosis

The analysis, as shown in Table 5, revealed that patients who developed gallstones experienced a mean absolute weight reduction of 37.62 ± 12.61 kg (median 35.50 kg, range 12.0-59.0 kg), corresponding to a percentage weight reduction of 30.80 ± 9.57% (median 31.51%, range 9.1-44.2%) from their pre-surgical weight.

Weight loss rate and time to gallstone diagnosis

**Figure 1:**
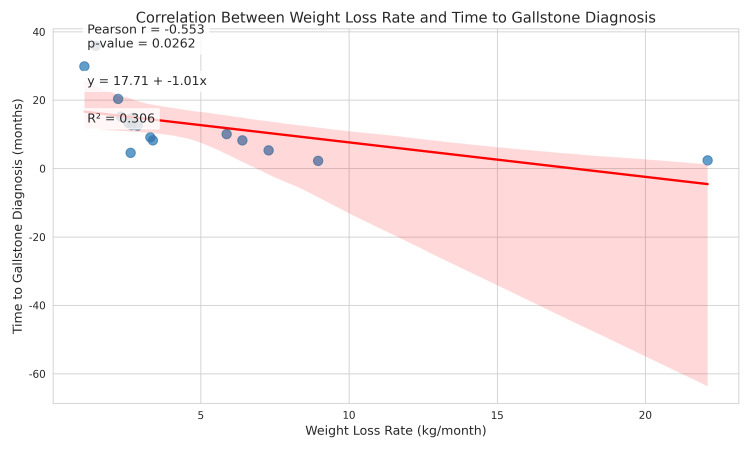
Pearson correlation analysis plot

The most compelling finding of this study was the relationship between the rate of weight loss and the timing of gallstone diagnosis. The mean rate of weight loss in patients who developed gallstones was 4.86 ± 5.11 kg/month (median 2.78 kg/month, range 1.1 - 22.1 kg/month), as can be noted in Table 5. Figure 1 shows that the data points illustrate a clear downward trend from left to right, which is summarized by the descending regression line. This indicates a negative correlation, indicating that as the rate of weight loss increases, the time to gallstone diagnosis tends to decrease (r = -0.553, p = 0.0262). Thus, patients with a faster rate of weight loss are strongly associated with a shorter time to the development of gallstone disease in this group of patients.

This relationship was further explored (Table 6) by categorizing patients into three groups based on the time to gallstone diagnosis: early (≤6 months), intermediate (>6 to ≤12 months), and late (>12 months). The median weight loss rates for these groups were 12.68 kg/month, 3.72 kg/month, and 2.21 kg/month, respectively. Kruskal-Wallis H-test was conducted to analyze the difference (p = 0.0045) in weight loss rates among the three gallstone diagnosis groups. This shows a statistically significant difference in the weight loss rate among the early, intermediate, and late gallstone diagnosis groups.

**Table 6:** Kruskal-Wallis test for the association of weight loss rate to diagnosis time

Diagnosis Group	Count	Median Weight Loss Rate (kg/month)	Mean Rank	H-Statistic	P-value
Early (≤6 months)	5	12.68	14	10.82	0.0045
Intermediate (>6 to ≤12 months)	4	3.72	8		
Late (>12 months)	7	2.21	4.86		

Following the significant result from the Kruskal-Wallis H-test, a post-hoc analysis was conducted to identify which specific groups have different weight loss rates. This was done by performing pairwise Mann-Whitney U tests with a Bonferroni correction to adjust the p-values for multiple comparisons. The post-hoc analysis confirms that the early diagnosis (≤6 months) group had a significantly faster rate of weight loss compared to both the Intermediate and late diagnosis groups. The weight loss rates between the intermediate and late diagnosis groups were not found to be significantly different from each other (Table 7).

**Table 7:** Post-Hoc test: pairwise Mann-Whitney U tests with a Bonferroni correction

Group 1	Group 2	Adjusted P-value	Significance
Early (≤6 months)	Late (>12 months)	0.008	Significant
Early (≤6 months)	Intermediate (>6 to ≤12 months)	0.048	Significant
Intermediate (>6 to ≤12 months)	Late (>12 months)	0.491	Not Significant

Linear regression analysis (Table 8) demonstrated that the rate of weight loss accounted for approximately 30% of the variance in the time to gallstone diagnosis (R² = 0.306), suggesting that while weight loss rate is an important predictor, other factors also contribute to the timing of gallstone development. These findings highlight the clinical importance of monitoring weight loss dynamics, particularly in the early postoperative period, as a potential indicator of gallstone risk.

**Table 8:** Linear regression analysis results

Dependent Variable	Independent Variable	R²	F-statistic	p-value	Regression Equation
Time to gallstone diagnosis (months)	Weight loss rate (kg/month)	0.306	6.18	0.0262	y = 17.71 - 1.01x

## Discussion

Medical literature published between 2015 and 2025 shows significant variation in the reported occurrence of gallstones following bariatric surgery, including LSG. Most studies report the rate of symptomatic gallstones to be between 3% and 10% [2]. In our study population, we noted an incidence rate of 16.3% for gallstone formation after LSG. Research indicates that the overall incidence of gallstones, encompassing both symptomatic and asymptomatic cases, can range from 10.4% to as high as 52.8% within the first 6-12 months after surgery [7]; however, the incidence of symptomatic gallstones after LSG is generally reported to be lower. Specifically, a study examining 114 patients who underwent LSG found that 7% developed symptomatic gallstone disease within an 18-month follow-up period, with eight of the 114 patients experiencing symptoms requiring treatment [2]. This rate aligns with findings from most previous studies on the topic, which have reported similar occurrence rates. In another study, a 3.5% incidence of symptomatic cholelithiasis was observed among 711 patients who underwent LSG over two years [12]. A review of records for 177 patients who had LSG reported a 4.5% incidence of gallbladder disease, with some of these patients experiencing symptomatic gallstones manifesting as biliary colic or cholecystitis [10]. A higher incidence was reported in a study of 96 patients after LSG, where 22.9% developed symptomatic gallstones [13]. Furthermore, a study examining 268 patients post-LSG found that 14.9% developed symptomatic cholelithiasis, on average, 10.65 months after surgery [14]. A study conducted in Saudi Arabia reported a high prevalence of gallstones (51.6%) after LSG, but did not specify the rate of symptomatic cases [15]. Another study indicated that gallstones formed in 14.6% of patients in the postoperative period following LSG [16].

Our study's findings provide compelling evidence for the pivotal role of weight loss dynamics in gallstone formation following LSG. While patients who developed gallstones experienced substantial absolute weight reduction, this amount of weight loss was not statistically significant in predicting the timing of gallstone development. This suggests that merely losing a large amount of weight does not, by itself, determine when or if gallstones will form. In stark contrast, the rate of weight loss emerged as a critical and statistically significant predictor. A significant negative correlation and linear regression analysis indicate a rate that explains approximately 30% of the variance in diagnosis, suggesting that the faster a patient loses weight, the earlier gallstones are likely to appear.

The variation in reported rates of symptomatic gallstone disease following LSG can arise from several factors. These factors include differences in study design, such as whether past data were analyzed or patients were followed over time, the number of patients involved, and the specific criteria used for including or excluding patients [7]. Variations in patient characteristics, such as gender, age, ethnicity, BMI before surgery, and the presence of other health conditions, may also affect the incidence of gallstone disease [2]. Furthermore, the follow-up duration for patients after surgery varied across studies, which can impact the reported rates since gallstones may develop at different times [7]. The definition used to classify a case as "symptomatic" gallstone disease might also differ between studies, potentially affecting the reported numbers [17]. Lastly, whether preventive measures, such as the regular use of ursodeoxycholic acid (UDCA), were employed by the study population can significantly influence the observed incidence of gallstone formation and subsequent symptomatic disease [2].

Our study has several limitations, including a small sample size, which restricts the statistical power and reliability of some findings. The exclusion of asymptomatic cases also underestimated the true incidence of gallstone formation, which could affect the comparative analyses. Being unable to account for other confounding factors may also influence study results, as these factors can impact both weight loss patterns and gallstone formation.

The dilemma of prophylactic cholecystectomy during LSG

The question of whether to routinely perform prophylactic cholecystectomy during the time of LSG in patients who do not have symptomatic gallstones before the surgery remains a significant clinical challenge. There are strong arguments on both sides of this issue.

Those who support routine prophylactic cholecystectomy argue that it can effectively prevent the future development of symptomatic gallstone disease and its related complications. By removing the gallbladder during the initial bariatric surgery, patients can potentially avoid the need for a second surgical procedure if they develop symptomatic gallstones later [7]. This approach could prevent complications such as biliary colic, acute cholecystitis, and pancreatitis, which can lead to significant illness, hospital stays, and further medical treatments. Furthermore, some suggest that while it adds to the initial cost of the bariatric surgery, prophylactic cholecystectomy might be more cost-effective in the long run by preventing the expenses associated with treating gallstone-related problems, including emergency room visits, hospital admissions, and subsequent cholecystectomies [18]. The main idea behind this argument is the potential to avoid future health issues and the associated healthcare costs by addressing a known risk factor at the time of the primary surgery.

On the other hand, there are compelling reasons against the routine performance of prophylactic cholecystectomy during LSG. One of the main concerns is the increased time required for the surgery when cholecystectomy is added, which could potentially increase the risk of complications during or shortly after the operation, such as bleeding, infection, and injury to nearby structures [16]. Moreover, most patients who undergo LSG may not develop symptomatic gallstone disease [2]. With most studies reporting that the incidence of symptomatic gallstones after LSG is below 10%, routine prophylactic cholecystectomy would expose a large number of patients to an unnecessary surgical procedure with its inherent risks. Additionally, LC is a well-established and safe surgical procedure that can be performed effectively if a patient develops symptomatic gallstones after LSG [14]. Some evidence suggests that performing cholecystectomy after the patient has lost some weight following LSG might even be technically easier due to the reduction in fat around the organs and liver [19]. Therefore, many argue that a selective approach, where cholecystectomy is performed only if symptoms arise, is more appropriate than a routine prophylactic strategy.

A review of the existing literature on the topic did not recommend routinely imaging the gallbladder before LSG. It did not support performing cholecystectomy at the time of LSG in patients without symptoms [16]. The evidence from these studies generally suggests that a selective approach to cholecystectomy in LSG patients without pre-existing symptomatic gallstones is more appropriate than a routine prophylactic approach.

## Conclusions

One major postoperative complication after LSG is symptomatic gallstone disease. This study underscores the clinical significance of gallstone disease as a postoperative complication and suggests potential approaches for risk stratification and monitoring. The study's most compelling finding is that the weight loss rate, rather than total weight, is a critical and statistically significant predictor of gallstone formation, especially for early onset, even though female sex remains a strong predisposing factor. This can serve as an important clinical marker for risk stratification and potential prophylactic intervention. A critical window for possible intervention is highlighted by the median time to gallstone diagnosis, which is approximately a year after surgery. The dilemma of prophylactic cholecystectomy in bariatric patients undergoing LSG remains a contentious issue, with good reasons for and against its widespread use. The potential benefits of routine prophylactic cholecystectomy need to be weighed against the risks and costs of an additional procedure. 

Given our findings, patients experiencing very rapid weight loss, particularly females, may warrant closer monitoring or consideration for prophylactic interventions. However, more extensive research is needed to establish definitive criteria for such interventions. Future research directions should include larger prospective studies with standardized follow-up protocols, the inclusion of both symptomatic and asymptomatic gallstone cases, and consideration of additional variables, such as the rate of weight loss, dietary factors, and genetic markers. Such comprehensive approaches would further elucidate the complex pathophysiology of post-bariatric surgery gallstone disease, inform evidence-based preventive strategies, and optimize patient management strategies. High-quality research in this area can lead to a more effective selection of patients for prophylactic cholecystectomy and the development of more personalized care plans following surgery.
